# Building Fucoidan/Agarose-Based Hydrogels as a Platform for the Development of Therapeutic Approaches against Diabetes

**DOI:** 10.3390/molecules28114523

**Published:** 2023-06-02

**Authors:** Lara L. Reys, Simone S. Silva, Diana Soares da Costa, Luísa C. Rodrigues, Rui L. Reis, Tiago H. Silva

**Affiliations:** 1 3B’s Research Group, I3Bs—Research Institute on Biomaterials, Biodegradables and Biomimetics of University of Minho, Headquarters of the European Institute of Excellence on Tissue Engineering and Regenerative Medicine, AvePark—Parque de Ciência e Tecnologia, Zona Industrial da Gandra, 4805-017 Barco, Guimarães, Portugal; larareys@gmail.com (L.L.R.);; 2 ICVS/3B’s—PT Government Associate Laboratory, 4710-057 Braga/Guimarães, Portugal

**Keywords:** fucoidan, agarose, hydrogels, diabetes mellitus, cell encapsulation, pancreatic beta cells, marine biomaterials

## Abstract

Current management for diabetes has stimulated the development of versatile 3D-based hydrogels as in vitro platforms for insulin release and as support for the encapsulation of pancreatic cells and islets of Langerhans. This work aimed to create agarose/fucoidan hydrogels to encapsulate pancreatic cells as a potential biomaterial for diabetes therapeutics. The hydrogels were produced by combining fucoidan (Fu) and agarose (Aga), marine polysaccharides derived from the cell wall of brown and red seaweeds, respectively, and a thermal gelation process. The agarose/fucoidan (AgaFu) blended hydrogels were obtained by dissolving Aga in 3 or 5 wt % Fu aqueous solutions to obtain different proportions (4:10; 5:10, and 7:10 wt). The rheological tests on hydrogels revealed a non-Newtonian and viscoelastic behavior, while the characterization confirmed the presence of the two polymers in the structure of the hydrogels. In addition, the mechanical behavior showed that increasing Aga concentrations resulted in hydrogels with higher Young’s modulus. Further, the ability of the developed materials to sustain the viability of human pancreatic cells was assessed by encapsulation of the 1.1B4HP cell line for up to 7 days. The biological assessment of the hydrogels revealed that cultured pancreatic beta cells tended to self-organize and form pseudo-islets during the period studied.

## 1. Introduction

One of the most promising areas of biomaterial development is the use of hydrogels as scaffolds for tissue engineering [1]. In this area, the application of hydrogels is very popular as these can act as a matrix for the delivery and retention of cells and growth factors within the site of injury, or as particles for encapsulation and sustained release of drugs [2,3,4,5]. In that way, different hydrogels have been proposed as versatile platforms for the engineering of bone, cartilage, vascular, and cornea tissue, as well as for the treatment of some diseases, namely diabetes mellitus [4]. Diabetes is a metabolic disorder characterized by high plasma glucose levels and can be classified as type I or type II (insulin-dependent or non-insulin-dependent, respectively). Pancreatic beta-cell replacement therapy by clinical islet transplantation (CIT) has been shown to be a promising alternative to conventional insulin treatment used for type I diabetes. However, CIT is limited by the significant risks involved in the use of immunosuppressive drugs to prevent the rejection of allogeneic donor islets. In diabetes monitoring and therapy, important cues have to be further explored, and thus advanced hydrogels are being developed to monitor high glucose levels, release insulin, or encapsulate pancreatic cells [6].

Hydrogels are insoluble 3D networks containing a high amount of water (at least 10% of the total weight or volume) [4,7], that may be produced using natural (i.e., agarose [8], collagen [9], gelatin [10], and chitosan [11]) and/or synthetic (i.e., poly (ethylene glycol) (PEG) [12], polyacrylamide (PAA) [13], polydimethylsiloxane (PDMS) [14]) polymers.

Hydrogels can be produced by physical gelation methods such as thermo-gelation [15], ionic crosslinking [16], pH sensitivity [17], and host–guest interaction [18] or by chemical crosslinking methods such as photopolymerization [19], click chemistry [20], enzyme-mediated crosslinking [21], and through the use of a crosslinking agent [22] establishing covalent bonds between polymeric chains to form an intricated network [23,24,25].

In this work, fucoidan and agarose were combined using thermo-gelation (changing from a fluid to a jelly-like state upon temperature decrease) to produce hydrogels as a platform with the potential to be applied in diabetes treatment. Fucoidan is a marine-origin sulfated anionic polysaccharide that can be extracted from different brown algae species, but also from some marine invertebrates (such as sea urchins and sea cucumbers (echinoderms)) [26,27,28,29]. Generally, the chemical structure of fucoidan is composed of l-fucose and sulfate groups, and different amounts of other sugars, such as glucuronic acid, xylose, galactose, and mannose, might also be present [27,29]. Fucoidan has been described as presenting diversified bioactive properties, such as anti-inflammatory [26,30], anticoagulant [26,31], antifibrotic [32], antibacterial, antitumor [31,33,34], blood glucose reducing [35], and antioxidant [7,31,36] activities. However, despite these promising biological properties, the biomedical use of fucoidan as a component of scaffolds faces some bottlenecks associated with its high water solubility hampering the production of cohesive polymeric matrices without chemical crosslinking strategies. Agarose, a neutral polysaccharide, is the main component of agar, which is derived from the cell wall of red algae [37,38]. The agarose structure consists of a basic repeat unit composed of 1,3-linked-d-galactopyranose and 1,4-linked 3,6-anhydro-α-l-galactopyranose [39,40]. Agarose can form gels in a thermo-reversible way under heating–cooling cycles with a significant hysteresis, suggesting its use as gelling agent suitable for many applications, including in the biomedical field, namely in the production of biomaterials for cartilage, nervous system, cardiac, bone, and corneal regeneration and diabetes treatment [41,42]. Considering in particular the latter, islet encapsulation in hydrogels has been studied for more than two decades as a potential treatment of type I diabetes mellitus, with the intent of keeping the implanted cells safe from the recipient´s immune system while keeping them active and producing insulin as needed [6,43]. Indeed, this type of scaffold can potentially provide an alternative to extra-hepatic transplantation site for islets by improving nutrient diffusion and blood supply to the scaffold, while preventing contact of the encapsulated cells with the host immune cells [43,44,45,46]. Moreover, several strategies have been explored, from simple hydrogel systems, with variable performance regarding the diffusion of oxygen and insulin depending on the gelling process, to functionalized systems incorporating growth factors promoting the formation of new vessels or smart responsiveness to external stimuli with an impact on permeability.

This work aims to create an innovative approach that associates marine biopolymers, agarose/fucoidan (AgaFu), using thermo-gelation. This innovative approach would promote the design of hydrogels with distinct features for future applications in diabetes treatment. To our knowledge, no reports in the literature suggest the use of agarose/fucoidan blends for biomedical applications, and naturally, there are no such reports on the specific area of diabetes treatments. With this purpose, marine biopolymers and the developed AgaFu hydrogels were extensively characterized regarding their structural changes, rheology behavior, and polymer distribution. The in vitro cytocompatibility evaluation of the developed AgaFu hydrogels was performed by encapsulating human pancreatic cells (1.1B4HP), and proliferation, morphology, and viability were investigated to potentiate their further use in diabetes treatment.

## 2. Results and Discussion

Functional hydrogels envisioned for use in diabetes treatment were developed by combining two marine-origin polymers, namely agarose (Aga), a gelling polymer, and fucoidan (Fu), a biologically active polymer, using the experimental conditions displayed in Table 1, with the respective formulations being designated as AgaFu #x, where the number in # represents the concentration of fucoidan in the solution, and the letter in x distinguish the different Aga/Fu weight ratios used (4:10; 5:10, and 7:10 wt).

**Figure 1:**
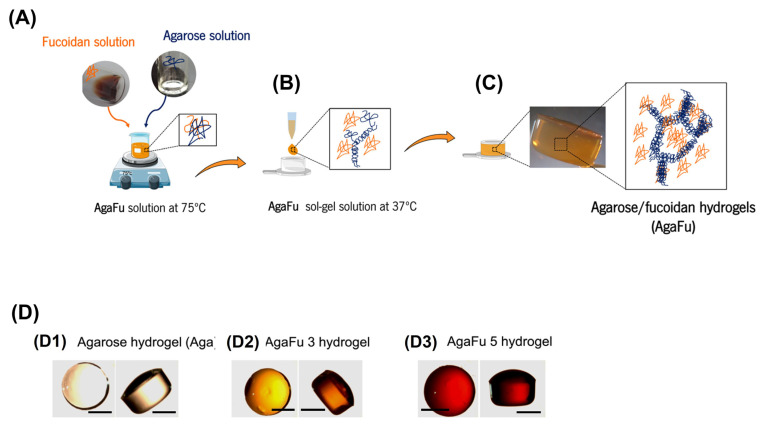
Schematic representation for production of agarose–fucoidan hydrogels (AgaFu). (**A**) Preparation of agarose (Aga) or agarose/fucoidan (AgaFu) solutions; (**B**) transferring of Aga or AgaFu solutions to molds; (**C**) Aga or AgaFu gel formation by cooling the solution on air until room temperature and AgaFu hydrogels; (**D**) photographs of the agarose/fucoidan-based hydrogels (scale bars: 5 mm): (**D1**) agarose hydrogel, (**D2**) AgaFu 3 hydrogel, and (**D3**) AgaFu 5 hydrogel.

The gel formation was obtained according to the representative scheme in Figure 1A–D. The solutions containing both fucoidan and agarose had a thermogelling behavior due to the action of agarose, being liquid at 75 °C but forming a gel upon temperature decrease until 37 °C. The blended hydrogels composed of AgaFu 3, and AgaFu 5 had a yellowish-brown color, good homogeneity, and mechanical stability (Figure 1A–C). Aga hydrogels (Figure 1(D1)) were used as control material rather than Fu alone; Fu does not form biomechanically stable structures (unless extensive crosslinking strategies are employed) due to its high water solubility [26,47].

### 2.1. Physical–Chemical Characterization of Fu, Aga, and AgaFu

To evaluate the influence of the composition of hydrogels on their physical–chemical properties, several characterization procedures were employed, and the obtained results were as follows.

#### 2.1.1. Rheology

**Figure 2:**
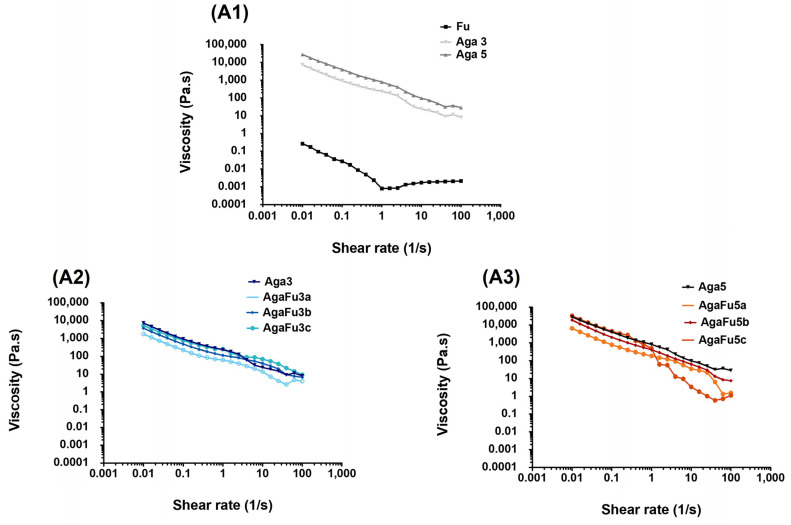

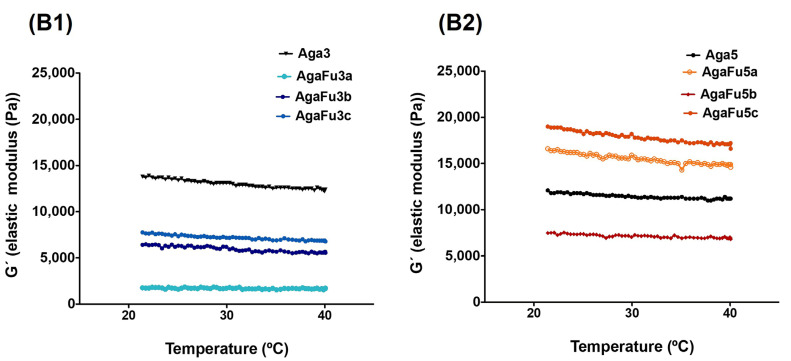

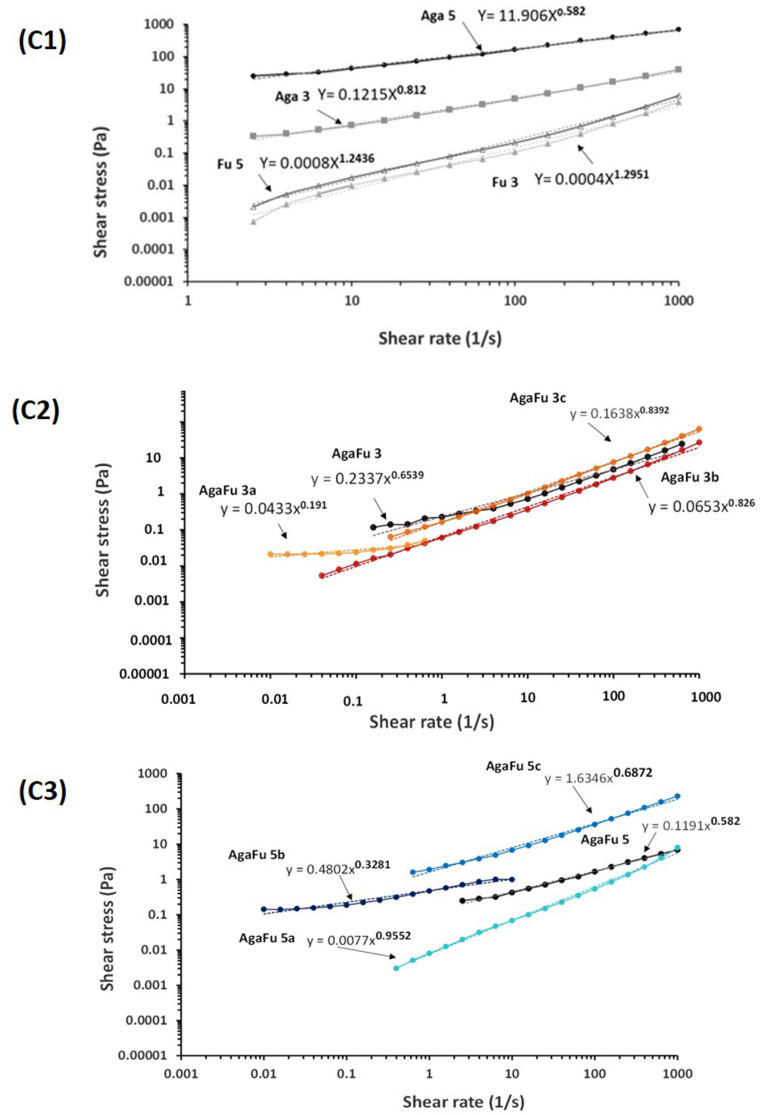
Rheological characterization of Aga 3, Aga 5, AgaFu 3, and AgaFu 5 hydrogels with different agarose:fucoidan weight ratios (a (4/10), b (5/10), and c (7/10)). (**A1**–**A3**) Viscosity curves as a function of shear rate, (**B1**,**B2**) oscillatory curves as a function of temperature, and (**C1**–**C3**) shear stress curves as a function of shear rate with adjusted power-law equations.

The viscosity of the solutions of Fu and Aga at 3 and 5% and the blended solutions (AgaFu 3 and AgaFu 5 at proportions a (4:10), b (5:10), and c (7:10)) was evaluated as a function of the applied shear rate (Figure 2(A1–A3)). As expected, the Fu solution presented lower viscosity when compared with Aga solutions, where differences in viscosity are directly correlated with the gelling property of Aga and its concentration. This reveals that the gel formation was fundamentally governed by the gelation of Aga, which can confine the fucoidan in the network established due to polymer chain rearrangement. Furthermore, both blended (AgaFu 3 and AgaFu 5) solutions displayed shear thinning behavior, in which the viscosity decreased with increasing shear rate (Figure 2(A2,A3)). This behavior was explained as the shearing of the polymer chains disrupting the three-dimensional structure by breaking primary and secondary bonds [48].

The prepared polysaccharide solutions were viscoelastic materials exhibiting solid and liquid characteristics. The storage (G′) and loss (G′′) moduli refer to the elastic and viscous response of a given material to shear stress, respectively [49,50]. In Figure 2(B1,B2), the evaluation of the storage modulus as a function of temperature is shown for Aga hydrogels of different concentrations (3 and 5%) and AgaFu 3 and AgaFu 5 at different proportions (a (4:10), b (5:10), and c (7:10)). All the curves show almost the same behavior. For the AgaFu 3 hydrogels, the higher ratio of Fu promoted a decrease in G′, with the storage modulus being independent of temperature (pseudo-equilibrium modulus), but the increase in Aga proportion in the blend increased the storage modulus for lower temperatures [48]. Furthermore, for the AgaFu 5 formulation, the same tendency was not so evident. The use of a more concentrated polymeric solution, with increased viscosities, particularly for the Aga solution, led to a more difficult interaction between the polymeric chains through a reduction in the chain mobility, also reducing the incidence of the intermolecular interaction. This is particularly noted for AgaFu 5b, where a significant decrease in G’ was observed when compared with AgaFu 5a. This difficulty was overcome for AgaFu 5c, where the substantial increase in the number of Aga molecules to pair and able to form the network of super-helices linked at their ends suppressed the influence of the reduction in the intermolecular interaction in the network [51].

The fitting of the experimental data (shear stress–shear rate) is presented in Figure 2(C1–C3) based on the power law model, y = k × x^n^, where y is the shear stress (Pa), x is the shear rate (1/s), k is the consistency coefficient (Pa·s^n^), and n is the flow behavior index, which describes the flow behavior of each solution [52,53]. In the literature, the flow behavior is classified according to this flow behavior index, and a Newtonian liquid presents n = 1, a pseudoplastic fluid presents n < 1, and a swelling plastic fluid presents n > 1. The Fu solutions, Fu 3% (n = 1.06 ± 0.03) and Fu 5% (n = 1.09 ± 0.06) solutions, presented a flow behavior index closest to 1, which is characteristic of Newtonian fluids, while Aga solutions, as well as most of the blended solutions, exhibited lower index values (AgaFu 3b (n = 0.826 ± 0.04); AgaFu 3c (n = 0.839 ± 0.04); AgaFu 5a (n = 0.955 ± 0.04)), suggesting a shear-thinning (pseudoplastic) flow behavior [54,55]. The results suggested that the shear-thinning behavior was governed by agarose [56]. The shear-thinning behavior, i.e., viscosity significantly decreasing with an increase in shear rate, suggests that the gel mixture of agarose and fucoidan could be injected by applying a high shear rate during injection with minimal effect on encapsulated cells and quickly self-heal after the removal of shear. Most significantly, these gels could be used to deliver biological molecules and cells during the injection process [56,57].

#### 2.1.2. Chemical Characterization

**Figure 3:**
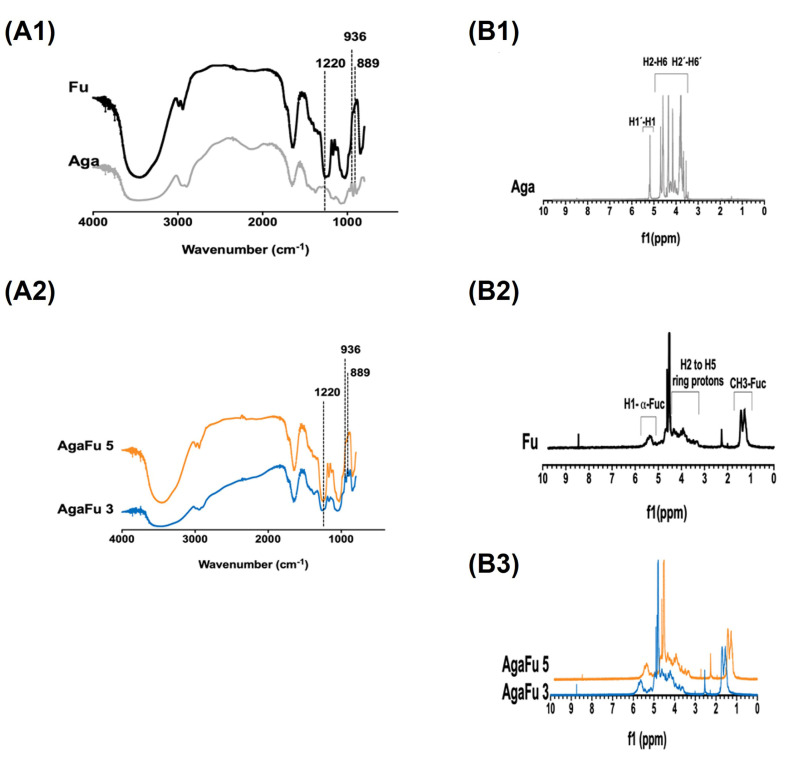
Chemical characterization of agarose (Aga), fucoidan (Fu), and produced hydrogels. FTIR spectra of (**A1**) agarose and fucoidan and (**A2**) AgaFu 3 and AgaFu 5 hydrogels. ^1^HNMR spectra of (**B1**) agarose, (**B2**) fucoidan, and (**B3**) AgaFu 3/AgaFu 5 hydrogels.

The chemical structures of the samples were examined by Fourier transform infrared spectroscopy (FTIR) (Figure 3(A1,A2)) and with proton nuclear magnetic resonance (^1^H NMR) spectroscopy (Figure 3(B1–B3)). On the FTIR spectra presented in Figure 3(A1), the main characteristic peaks of Aga and Fu can be observed. On the Aga spectra, the region located between 900 and 1200 cm^−1^ contains the characteristic saccharide peaks of Aga that are representative of the skeletal vibrations associated with C-O-C glycosidic bonds. The region 890–940 cm^−1^ also contains a characteristic signal of Aga that represents vibration associated with the C-O-C bridge on the 3,6-anhydrogalactose unit [55,58]. FTIR spectra of fucoidan showed an absorbance peak at 1000–1270 cm^−1^, corresponding to the sulfur–oxygen double bond (S=O) stretching vibration of the sulfate group. The spectra also showed signals in the ranges of 1315−1220 cm^−1^ and 1140–1020 cm^−1^, assigned to the symmetric and asymmetric stretching of ether sulfate groups (RO−SO_3_−) [26,46,59]. A broad band around 3200–3500 cm^−1^ also appears due to the stretching of –OH groups, a characteristic of carbohydrate monomers [26,55,60]. The characteristic peak of C-O-S stretching at 845 cm^−1^ was also attributed to the presence of sulfate groups from fucoidan [61]. For the further chemical characterization of AgaFu hydrogels, composition b (AgaFu 3b and AgaFu 5b) with an intermediate wt ratio of agarose (Aga) and fucoidan (Fu) was used.

Moreover, all spectra of AgaFu hydrogels presented in Figure 3(A2) showed an absorbance band at 1220 cm^−1^, corresponding to the sulfate groups characteristic of Fu [26,60]. This indicates that the intrinsic sulfate group of Fu is also present in the AgaFu 3 and AgaFu 5 formulations after the blending with Aga, confirming the presence of fucoidan in the final hydrogels, which is in accordance with what was already suggested by their brownish color. In FTIR spectra of both AgaFu 3 and AgaFu 5, we observed bands characteristic of Aga and Fu, suggesting that interactions between the polymers could occur, as discussed in the rheological characterization.

Furthermore, proton nuclear magnetic resonance (^1^H NMR) spectroscopy of Aga showed two groups of peaks, one at δ = 5.15–5.25 ppm corresponding to protons C1 and C1′ from the Aga skeleton and the other at δ = 3.45–4.61 ppm, attributed to protons C2-6 and C2′-6′ in the Aga skeleton (Figure 3(B1)). Fu ^1^H NMR spectra showed different peaks at δ = 1.20–1.30 and 1.95–2.26 ppm corresponding to methyl groups of fucose and l-fucopyranose; at δ = 3.35–4.55 ppm, confirming the presence of different types of fucose groups with changes in glycosidic linkage positions and monosaccharide patterns; and at δ = 5.29–5.40 ppm, corresponding to anomeric protons of the l-fucopyranosyl unit (Figure 3(B2)) [46,62]. The spectra corresponding to AgaFu 3 and AgaFu 5 solutions (Figure 3(B3)) showed peaks characteristic of Aga and Fu, suggesting the existence of interactions between the polysaccharides. Additionally, the results obtained were compared in the literature with other systems that use these polymers, such as fucoidan, and it is verified that peaks appear at the same chemical shift (δ) values [61,63].

#### 2.1.3. X-ray Photoelectron Spectroscopy (XPS)

**Figure 4:**
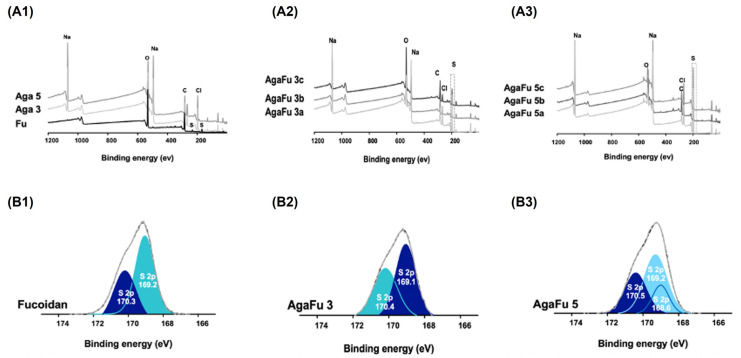
XPS wide scan of (**A1**) Fu (powder) and Aga 3 and Aga 5 hydrogels, (**A2**) AgaFu 3 hydrogels, and (**A3**) AgaFu 5 hydrogels with different agarose:fucoidan weight ratios (a (4/10), b (5/10), and c (7/10)). XPS images of element distribution, particularly the sulfur region, of (**B1**) Fu (powder), (**B2**) AgaFu 3 hydrogels, and (**B3**) AgaFu 5 hydrogels.

The superficial chemical composition of the AgaFu hydrogels was also examined by XPS surface measurements (Figure 4A,B). The XPS data are in accordance with the data obtained by ^1^H NMR and FTIR. The XPS wide spectra of Fu, Aga, and the hydrogels prepared in different proportions (AgaFu 3 and AgaFu 5) displayed the presence of the expected peaks of carbon (C), oxygen (O), calcium (Ca), sodium (Na), and sulfur (S) (Figure 4A,B). In addition, the surface atomic ratios of the main components (S2p, O1s, and C1s) and the respective ratios of O/C and S/C of all samples analyzed are represented in Table 2. As expected, the amount of sulfur is higher in the samples with Fu, because fucoidan contains sulfur in its atomic composition, namely in sulfate groups. No obvious differences were noted in the C1s and O1s region spectra of the AgaFu hydrogels (Figure 4(B2,B3)) when compared with the same regions for the isolated Aga and Fu separately. The increase in Aga concentration in the hydrogel formulation led to a decrease in the presence of sulfur (S) in the sample, as the ratio of fucoidan decreased. Similar behavior was observed when Fu concentration increased in the blended hydrogel: there was an evident increase in the S/C ratio. This result is in accordance with previously reported chemical characterization (FTIR, ^1^HNMR) where Aga and Fu characteristic peaks appeared in the spectra of AgaFu samples, thus confirming the blending of both polymeric components.

#### 2.1.4. Mechanical Properties and Hydrogel Mesh Size

**Figure 5:**
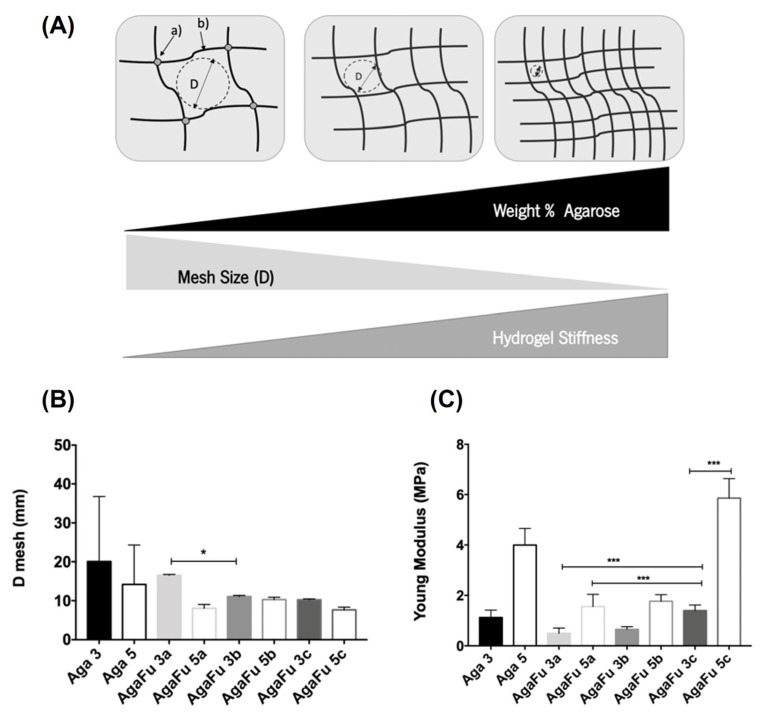
(**A**) Schematic representation of a hydrogel network consisting of (a) connective crosslinks and (b) polymer chains with average mesh size D. (**B**) Mesh size and (**C**) Young’s modulus of Aga 3, Aga 5, AgaFu 3, and AgaFu 5 hydrogels with different agarose:fucoidan weight ratios (a (4/10), b (5/10), and c (7/10)) (data represent means ± standard deviation; * *p* < 0.05, *** *p* < 0.001, one-way ANOVA).

The mesh size of the hydrogel was calculated based on the equivalent network model Equation (1) (please see Section 3.4.2 below). Figure 5B shows that when Aga concentration increased, there was a decrease in the mesh size, attributed to tightly packed Aga polymeric chains [38,64] (Figure 5A). Agarose is soluble in water at temperatures between 85 °C and 100 °C [41]. So, in this work, the Aga solution was heated up to 85–95 °C, which provided sufficient energy to break the hydrogen bonds, and the Aga chains were oriented with no long-range order [38,59,65]. However, as the temperature decreases and hydrogen bonds start to form, Aga molecules pair up into double helices, which themselves aggregate with other double helices to form thicker fibers. The gel is formed due to this network of super-helices linked at their ends [38,59,65]. The results also showed that the mesh size of hydrogel blends decreased with the increase in the concentration of Aga in the solution from 3 to 5%. This may be related to an increase in the density of the initial solution and consequently of the produced hydrogel. Moreover, independently of the polymeric proportion studied, it is possible to note that the combination of Aga with Fu promoted a reduction in the mesh size of the produced hydrogel. This fact should be related to the intermolecular interactions established between Aga and Fu polymeric chains (e.g., electrostatic bonds, hydrogen bonds, ion–dipole force, and London forces) that allow the formation and maintenance of the blended network, overcoming the natural non-gelling ability of Fu. Considering the blended hydrogels prepared using a lower initial concentration of Aga (3a, 3b, and 3c), a decrease in the mesh size is observed with the reduction in the Fu proportion in the blended mixture or an increase in the Aga proportion. Besides the existent intermolecular interactions, the increasing number of Aga molecules to pair promotes an increase in the density of the hydrogel, which leads to more tightly packed helices, consequently promoting a decrease in the mesh size [38,59]. Furthermore, when comparing the hydrogels prepared using the same polymeric ratio and different concentrations of Fu solution, a decrease in mesh size is noted for the hydrogels prepared with the highest concentration of Fu; however, there are no significant differences.

Young’s modulus of AgaFu hydrogels seemed to increase with increasing Aga and Fu concentration, although statistically significant differences between formulations have been identified only for samples AgaFu 3a, AgaFu 3c, AgaFu 5a, and AgaFu 5c (Figure 5C). This result correlates well with the mesh size results, with the decrease in the mesh size being associated with an increase in the stiffness of the hydrogels, as represented schematically in Figure 5A [54].

#### 2.1.5. Diffusion Tests and Permeability to Glucose

**Figure 6:**
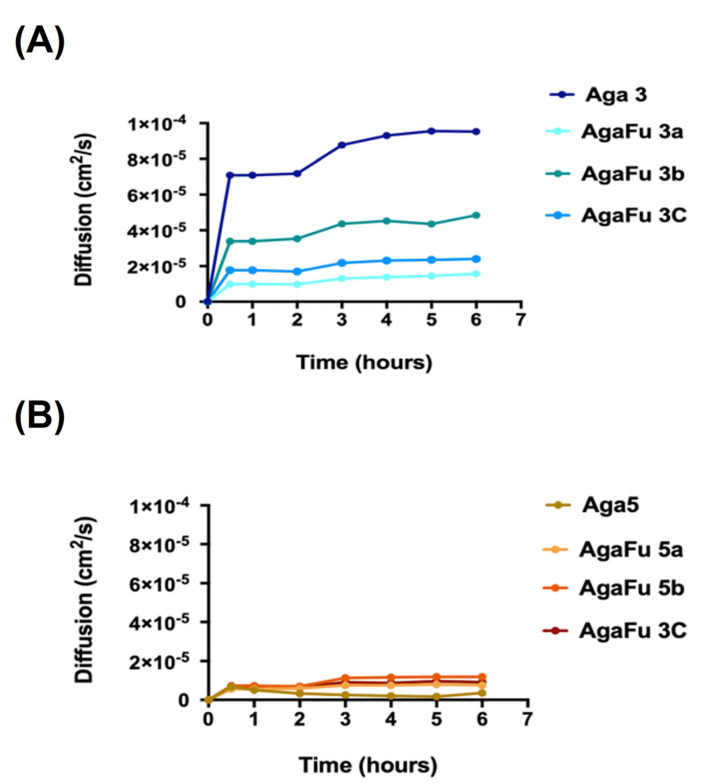
Diffusion of glucose across (**A**) Aga 3 and AgaFu 3 and (**B**) Aga 5 and AgaFu 5 hydrogels with different agarose:fucoidan weight ratios (a (4/10), b (5/10), and c (7/10)).

The methylene blue assay was carried out as a preliminary assessment of the diffusion of compounds within the developed hydrogels [66]. The diffusion of methylene blue through the Aga and AgaFu hydrogels showed that samples with a higher concentration of agarose have a slower diffusion of methylene blue, caused by a denser matrix as suggested by the reduced mesh size. However, the diffusion of methylene blue through the hydrogel blends, AgaFu, seems to be hampered when compared with pure Aga hydrogels, which may suggest a stronger interaction between the polymers and consequently more cohesive and compact systems, particularly for the samples AgaFu 3/5c where methylene blue seemed to exhibit the slowest diffusion [66]. To complement the information obtained from the evaluation of the diffusion of methylene blue, the permeability of AgaFu hydrogels to nutrients was also assessed using glucose as a model molecule for permeation. Diffusion profiles showed an initial lag phase in the receptor cell due to the time required for the solute to traverse the membranes [67,68] (Figure 6). After this phase, the bulk liquid reached equilibrium after approximately 1 h. The higher water content of native membranes facilitates the permeation of water-soluble molecules such as glucose, which is related to the mesh size of the hydrogel [67,69]. The permeability coefficient (P) was determined using Equation (2) (please see Section 3.4.8 below), and the obtained values increased when the fucoidan ratio increased, as shown in Table 3. These results could be related to the charge of the compounds, their hydrophobicity, and the mesh size of the hydrogel. The permeability tends to increase with anionic compounds and the ones that have a higher affinity with water [70]. The hydrogels that have smaller mesh sizes and are more cohesive and compact systems tend to have reduced permeability, as discussed and demonstrated with methylene blue. The diffusion coefficient (D) values were deduced from Equation (3) (please see Section 3.4.8 below), and the partition coefficients were deduced from Equation (4) (please see Section 3.4.8), with the obtained data being depicted in Table 3. The diffusion coefficient is a crucial parameter for estimating the mass of a solute diffusing in time through the gel [49]. The obtained data showed that the increase in the Aga concentration resulted in lower diffusion coefficients, in accordance with the permeability observations and the more cohesive and compact systems obtained in these conditions. These observations are in accordance with the results reported by Marchioli et al. [43] when seeing an impact of scaffold porosity on performance, with porous alginate scaffolds showing a better permeability to nutrients than bulk hydrogels due to a higher surface-to-volume ratio. Moreover, Farina et al. [44] also highlighted the impact of the thickness of capsules on the transport of oxygen and, consequently, on cell viability in cell-encapsulating devices. Furthermore, it is known that permeability is also directly related to immune protection, which is associated with the interaction and diffusion of antibodies and the need to avoid contact between a graft and host cells [71]. The partition coefficient (K_d_) is a measure of solubility in the hydrogels [69], and it increased with increasing fucoidan concentration (data in Table 3), which is attributed to a higher water uptake capacity and, consequently, the solutes having a higher ability to remain trapped within the hydrogel [67].

### 2.2. Biological Characterization

The biological performance of the Aga and AgaFu hydrogels was tested using the human pancreatic beta-cell line 1.1B4HP. The 1.1B4HP cells have been used in several studies focusing on pancreatic beta-cell biology, representing a source of cellular therapy for type 1 diabetes [72]. To evaluate the effect of using fucoidan, we have compared the performance of AgaFu hydrogels with Aga hydrogels rather than Fu alone, as Fu does not form 3D-based structures alone due to its high water solubility [26,47,73]. To assess if encapsulated 1.1B4HP cells were able to proliferate while maintaining viability in Aga and AgaFu hydrogels, cell-laden samples were analyzed after 1, 3, and 7 days of culture by MTS assay, DNA quantification, live/dead assay, and evaluation of cell morphology by confocal laser scanning microscopy (CLSM).

**Figure 7:**
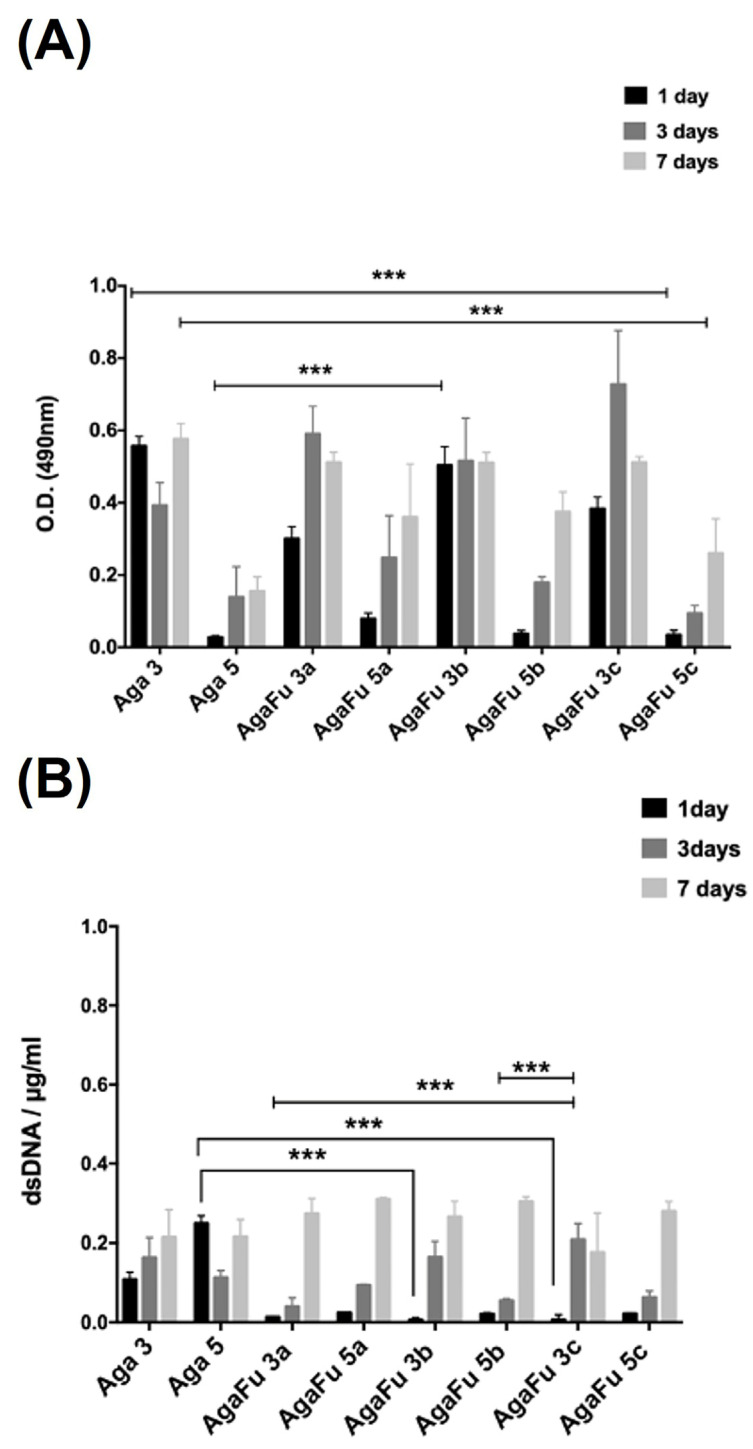
(**A**) MTS results and (**B**) dsDNA content of 1.1B4 human pancreatic cells encapsulated in Aga 3, Aga 5, AgaFu 3, and AgaFu 5 hydrogels with different agarose:fucoidan weight ratios (a (4/10), b (5/10), and c (7/10)), during up to 7 days in culture (data represent means ± standard deviation; *** *p* < 0.001, Kruskal–Wallis test).

The MTS results showed, in general, an increase in 1.1B4HP cells’ metabolic activity during the culture time in both Aga and AgaFu hydrogels (Figure 7A). Moreover, comparing the results obtained for hydrogels prepared with different polymeric concentrations (agarose solutions at 3% or 5%), we observed that 1.1B4HP cells had lower metabolic activity in the samples with higher Aga concentrations. In addition, results obtained from dsDNA quantification did not show relevant differences in the proliferation of 1.1B4HP cells encapsulated in Aga and AgaFu hydrogels (Figure 7B). These results are correlated with the mesh size of the hydrogel, as the increase in Aga and Fu concentrations would reduce the mesh size of the hydrogel, and the cells would be much more space-constrained and potentially face lower nutrient diffusion, causing slower metabolic activity. In fact, Strand et al. have pointed to the variation in oxygen concentration through the encapsulation devices, depending on the shape and surface-to-volume ratio, and its impact on cell viability [71].

**Figure 8:**
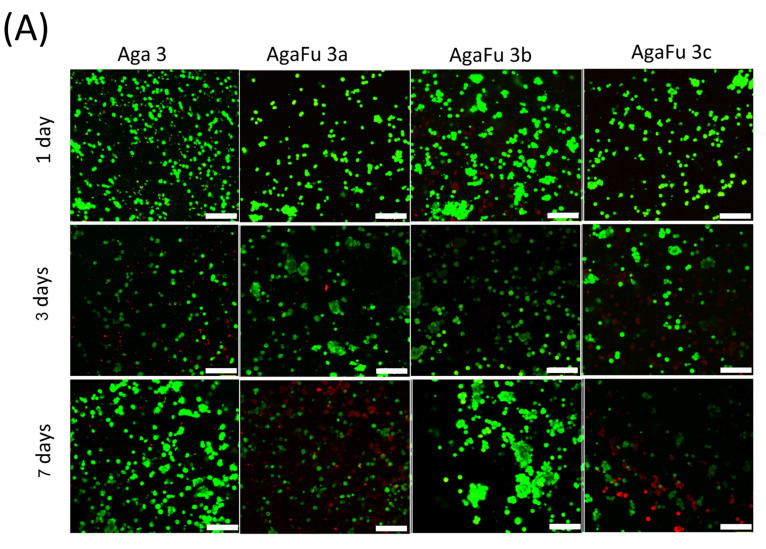

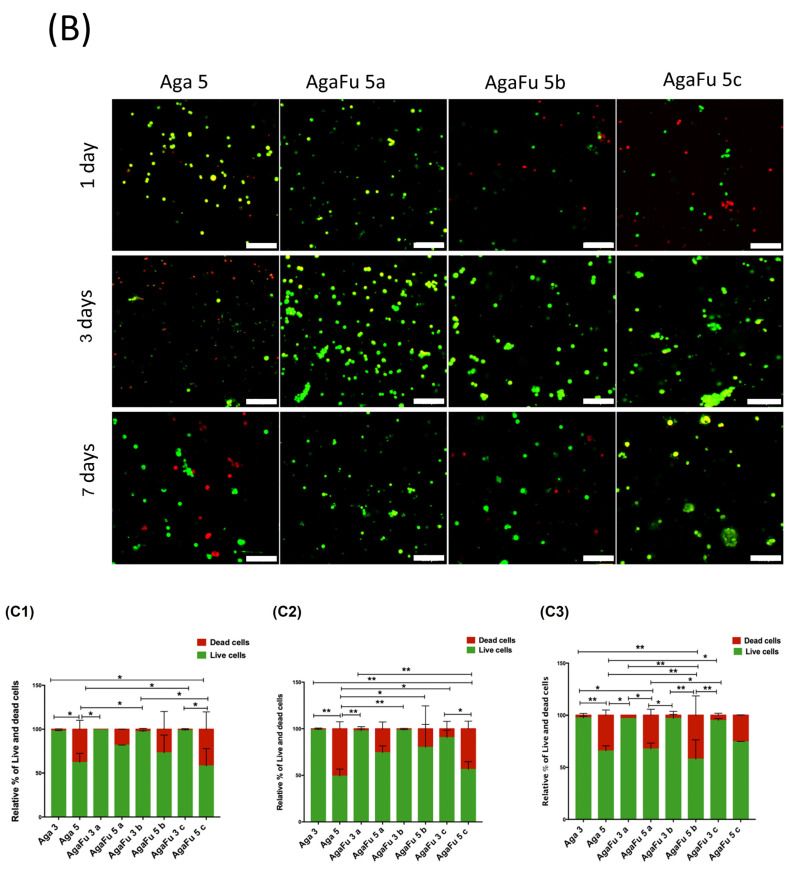
Live/dead microscopy images of 1.1B4HP cells encapsulated into (**A**) Aga and AgaFu 3 and (**B**) Aga and AgaFu 5 hydrogels with different agarose:fucoidan weight ratios (a (4/10), b (5/10), and c (7/10)), after 1, 3, and 7 days in culture. The cells were stained with calcein/ethidium solutions; live cells (green) were calcein-positive, and dead cells (red) were ethidium-homodimer-positive; scale bar: 100 µm. (**C**) Quantification of cell viability in the different hydrogels after (**C1**) 1 day, (**C2**) 3 days, and (**C3**) 7 days in culture (data presented as mean ± standard deviation; * *p* < 0.05, ** *p* < 0.01).

The ability of hydrogels to support cell viability was also evaluated by a live/dead staining assay. Confocal images obtained after 1, 3, and 7 days of culture (Figure 8) allowed the conclusion that all the hydrogel formulations showed viable cells, demonstrating that the biomaterials proposed in this work are generally not cytotoxic (Figure 8A,B). Results obtained with hydrogels produced with lower polymer concentrations (3% agarose or 3% fucoidan and different ratios of agarose) showed higher rates of cell viability, while hydrogels produced with the highest polymeric content presented the lowest number and ratio of live cells (~45%), potentially translating the effect of more cohesive and compact hydrogels and consequently the spatial confinement of the cells, with a negative impact on cell viability. These results are in agreement with diffusion coefficient values, where an increase in the Aga concentration resulted in lower diffusion coefficients, which can be translated into a decreased access to nutrients for encapsulated cells, resulting in an increment in dead cells and a decrease in overall metabolic activity. The effect of hydrogel formulation is more evident than the one observed by Marchioli et al. [43] with alginate-based materials (as they detected the impact of the components of the hydrogel but, surprisingly, not on the shape as this would affect the surface-to-volume ratio and consequently the transport of oxygen and nutrients). Indeed, those authors attribute the observed limitations on cell viability to hampered nutrient transport, given the viscous and highly concentrated gel used in their work, thus suggesting the relevance of hydrogel mesh size for cell fate.

**Figure 9:**
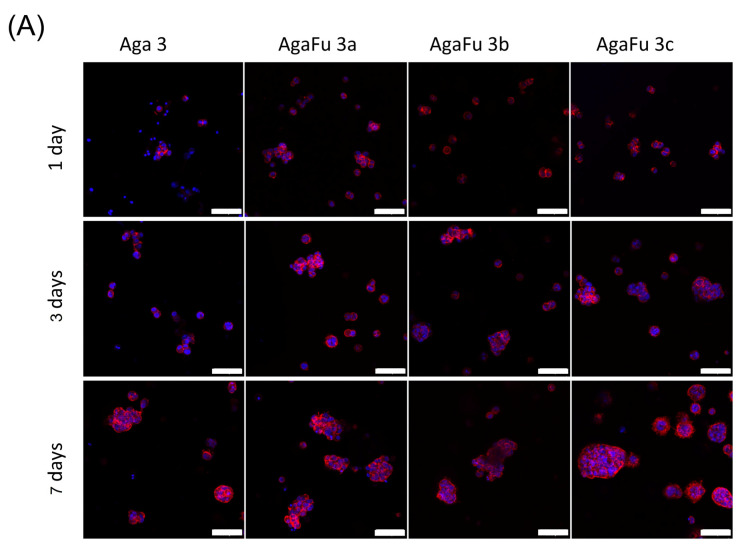

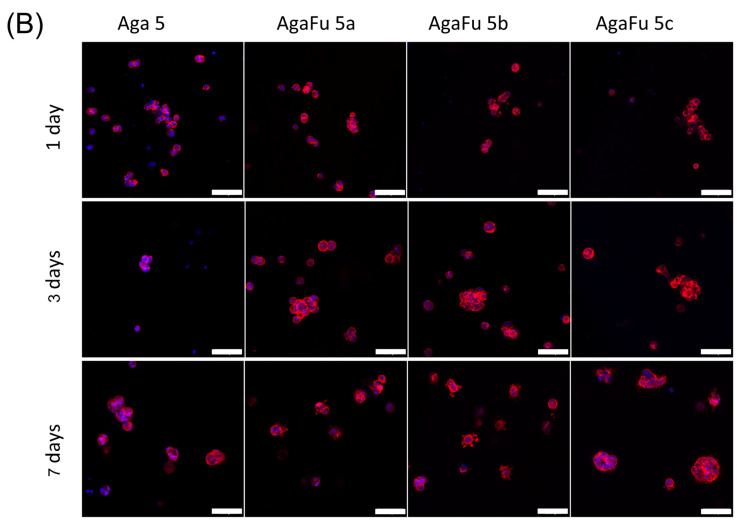
Confocal images of 1.1B4HP cells encapsulated in (**A**) Aga 3 and AgaFu 3 and (**B**) Aga 5 and AgaFu 5 hydrogels with different agarose:fucoidan weight ratios (a (4/10), b (5/10), and c (7/10)) for 1, 3, and 7 days in culture. Staining of actin (red) and nuclei (blue); scale bar: 100 µm.

The morphology of the encapsulated cells was evaluated by CLSM after the staining of actin (cytoskeleton) and nuclei with phalloidin and DAPI, respectively. The obtained images show that cells had a round-like shape and tended to self-organize, forming pseudo-islets after 3 days of culture (Figure 9A,B). This pseudo-islet morphology was maintained for up to 7 days of culture (Figure 9), which is characteristic of human pancreatic cells [74]. The noted behavior suggested that the formulated hydrogels with 3% fucoidan supported the encapsulation of 1.1B4HP cells that remained viable for at least 7 days (Figure 7 and Figure 8). Moreover, the presence of fucoidan seemed to allow a higher number of cells to be encapsulated in the hydrogels, enabling the self-organization of larger pseudo-islets.

## 3. Materials and Methods

### 3.1. Materials

Fucoidan (Fu) in powder from brown algae *Fucus vesiculosus* (Maritech Fucoidan, Marinova, FVF2011527, Tasmania, Australia) was used after a purification process as described below. Agarose with low gelling temperature for molecular biology (Sigma-Aldrich, MO, USA) was used as received. All other reagents were of analytical grade and used as received.

### 3.2. Purification of Fucoidan (PFu)

The fucoidan (Fu) powder was purified based on the calcium ion methodology [46]. The purification with calcium ions was performed to remove the alginates contained in the crude fucoidan. These alginate residues form complexes with calcium ions and precipitate [46]. Briefly, Fu was dissolved (160 mg mL^−1^) in a 20 mM calcium acetate aqueous solution for 4 h at 50 °C under stirring, and the pH was adjusted continuously between 6.6 and 7.5 to avoid acid hydrolysis of fucoidan. Afterward, the solution was left at 4 °C overnight and then centrifuged for 15 min at 5000× *g* (Centrifuge 5810, Eppendorf, Leipzig, Germany). The supernatant was dialyzed against water using 12–14 KDa cut-off dialysis membranes for 3 days and freeze-dried (CryoDos-80, Telstar, Terrassa, Barcelona, Spain) for 4 days [46].

### 3.3. Preparation of Fucoidan-Based Hydrogels

Agarose/fucoidan (AgaFu) hydrogels were prepared by thermo-gelation [38,59]. Purified fucoidan (Fu) was dissolved in ultrapure water at a concentration of 3 or 5% (*w*/*v*) under stirring at 75 °C, and then Agarose (Aga) powder was added to the solutions (Figure 1A). The AgaFu hydrogels were prepared with different Aga/Fu proportions (4:10; 5:10, and 7:10 wt) and represented as AgaFu 3X or AgaFu 5X (where different letters in X represent different formulations) throughout the manuscript, as indicated above in Table 1. Agarose has a thermogelling behavior, and at this temperature (75 °C) the formulations were in a liquid solution state. Each of the obtained solutions was then cast into Eppendorf caps (circular plastic with size 9.73 ± 0.09 mm width and 5.38 ± 0.09 mm height) that were used as molds, and the systems were left to cool to room temperature (~22 °C). With the decrease in temperature, the viscosity of the solutions increased due to the presence of agarose, and when room temperature was reached, hydrogels had been formed (Figure 1B,C). After being removed from molds and considering that they would be used for cell encapsulation, the hydrogels were replenished with Dulbecco´s phosphate-buffered saline (DPBS) solution (Figure 1D), which has a lower concentration of ions in comparison to standard PBS and was deemed necessary for the cohesion of the produced hydrogels. Aga hydrogels were used as control material rather than Fu alone, as Fu does not form structures (without the use of chemical crosslinking) due to its high water solubility [26,47]. The Aga hydrogels were produced with the same procedure but without fucoidan addition.

### 3.4. Physical–Chemical Characterization of AgaFu Hydrogels

#### 3.4.1. Rheology

Rheological assays were performed using a Kinexus pro+ rheometer (Malvern Instruments, Malvern, UK), with the acquisition software rSpace v1.7 (Malvern Instruments, Malvern, UK).

Oscillatory and rotational measurements: All the oscillatory measurements were performed using stainless-steel cone–plate geometry (40 mm, 4°), while rotational measurements were performed using a parallel plate geometry (1 mm gap), using solutions equivalent to those used to prepare hydrogels, and Aga and AgaFu formed hydrogels. In the rotational experiments, two different plots were obtained: (a) the traditional shear viscosity (Pa·s) as a function of shear rate (1/s) and (b) the shear stress (Pa) as a function of shear rate (1/s). From these curves, it was possible to obtain information about the flow behavior of the samples. The oscillatory experiments were preceded by the measurement of strain sweep curves to determine the linear viscoelastic region (LVER) of the samples. Strain amplitude was thus measured in the shear rate range changing from 0.1 to 100 Hz in a 10/decade rate, at a range of cooling temperatures between 80 °C and 37 °C and a constant 1% strain. After this, the mechanical spectra of each sample were obtained, and the elastic modulus (G′) and viscous modulus (G″) were recorded over a frequency sweep ranging from 0.1 Hz to 100 Hz at a constant temperature of 37 °C. The strain dependency measurements were performed using 1 mL of the solution or an Aga or AgaFu hydrogel (8.91 ± 0.09 mm width and 5.38 ± 0.09 mm height) in the sample holder [53,75]. All plots are the average of measurements with at least 3 different samples.

Compressive studies: These tests were performed using a parallel plate geometry of 8 mm diameter, with radial grooves to avoid hydrogel leakage. Aga and AgaFu hydrogels were prepared in distilled water as previously described and maintained in DPBS. Hydrogels were compressed in the direction normal to the circular face of the cylindrical samples at a rate of 1 mm/min for a maximum strain of 85% and at room temperature. The Young’s modulus was determined from the initial slope of the linear region of the stress–strain curve. Each experiment was performed in quintuplicate [54].

#### 3.4.2. Hydrogel Mesh Size

The mesh size is often described by a structural parameter, D_mesh_, which represents the diameter of a hard sphere that fills the void between hydrogel network strands. Hydrogel mesh size was calculated based on the classical theory of rubber elasticity, which relates the shear modulus (G′) to the mesh size D_mesh_ (Equation (1)):

(1) $$D_{mesh}={\left(\frac{6RT}{\pi N_{av}G\prime }\right)}^{\frac{1}{3}}$$

where R is the gas constant, T the absolute temperature, and N_av_ is Avogadro’s number. This allows the estimation of the mesh size of a hydrogel based on its shear modulus [76,77].

#### 3.4.3. ^1^H NMR Spectroscopy

Aga and Fu powders and AgaFu freeze-dried hydrogel powder were analyzed by ^1^H NMR spectroscopy, after being dispersed/dissolved in deuterated water (D_2_O) at 5 mg mL^−1^. The ^1^H NMR assay was performed at 50 °C using a Bruker Avance III according to the following spectral conditions: 300 Hz spectra with 90° impulses, 4 s acquisition time. Spectra were processed using Mestre Nova v14.0 software (Mestrelab Research S.L., Santiago de Compostela, Spain).

#### 3.4.4. Fourier Transform Infrared Spectroscopy (FTIR)

The FTIR analysis of the samples was performed on a Shimadzu IR Prestige 21 spectrometer (Shimadzu Corporation, Kyoto, Japan). Samples were prepared using potassium bromide (KBr) pellets at room temperature. Each sample spectrum was collected by averaging 32 scans with a resolution of 16 cm^−1^, within the 4000–400 cm^−1^ spectrum regions.

#### 3.4.5. X-ray Photoelectron Spectroscopy (XPS)

Analysis of the Aga, Fu, and AgaFu samples was performed using a Kratos Axis-Supra instrument (Kratos Analytical Ltd., Manchester, UK), equipped with an aluminum Kα monochromatized radiation X-ray source (1486.6 eV) and ESCApe software (1.5 version, Kratos Analytical Ltd., Manchester, UK). Due to the non-conducting nature of the samples, it was necessary to use a co-axial electron neutralizer to minimize surface charging, which performed the neutralization by itself. Photoelectrons were collected from a take-off angle of 90° relative to the sample surface. The measurement was performed in constant analyzer energy (CAE) mode with a 160 eV pass energy for survey spectra, 20 eV pass energy for high-resolution spectra of the hydrogels, and 40 eV pass energy for the polymer powders. Charge referencing was performed by setting the lower binding energy C1s peak at 285.0 eV related to the C1s hydrocarbon peak [53,78].

#### 3.4.6. Stability Test

Stability tests of the Aga and AgaFu hydrogels were performed by immersion in DPBS and in Roswell Park Memorial Institute (RPMI) 1640 medium supplemented with 10% fetal bovine serum (FBS) (Invitrogen, MA, USA) and 1% antibiotic/antimycotic (A/B) (Invitrogen, USA) for 28 days at 37 °C. The medium was replaced every 3 days. The swollen sample weight was measured after removing the excess surface water by gently tapping the surface with filter paper. The swelling ratio was given by the ratio W_s/_/W_d_, where W_s_ is the swollen sample weight under specified environmental conditions and W_d_ is the dry sample weight.

#### 3.4.7. Diffusion within Hydrogels

The diffusion of a model compound within Aga and AgaFu hydrogels was assessed based on the Kirby–Bauer method [79]. The AgaFu solutions were prepared as previously mentioned (preparation of fucoidan-based hydrogels). These solutions were dropped in a Petri dish, and gelation occurred at room temperature for 15–20 min. Afterward, some holes (d = 6 mm) were made in the formed AgaFu gels, and a 100 mM methylene solution was dropped into those holes. The diffusion of methylene blue is calculated by the difference between starting position of the dye and the diameter of the dye spot at different time points.

#### 3.4.8. Permeability to Glucose

The permeability to glucose was determined using a glass Franz-type diffusion cell (PermeGear) with an 8 mL reactor compartment (using an effective mass transfer area of 1 cm^2^) [67]. Aga and AgaFu hydrogels were previously equilibrated in a DPBS solution (overnight), placed between the two compartments, and held with a stainless-steel clamp. The receptor compartment was immediately filled with DPBS solution, and air bubbles were removed. Finally, the donor compartment was filled with 16 mM of glucose (DPBS, pH 7.4). Aliquots of 1 mL were removed from the receptor compartment at pre-established time points, and the compartment was further restored with the same quantity of fresh DPBS. The experiments were performed at 37 °C, and the receptor compartment was stirred using a magnetic bar to eliminate the boundary layer effect. The glucose concentration in the receptor chamber was evaluated using the dinitrosalicylic acid (DNS) method. DNS reacts with reducing sugars and other reducing molecules, forming 3-amino-5-nitrosalicylic acid, an aromatic compound that strongly absorbs light at 540 nm, enabling the determination of the amount of reducing sugar present in the solutions. The amount of glucose was calculated by measuring the absorbance at a wavelength of 540 nm using a microplate reader (Synergy HT, Bio-TEK, Santa Clara, CA, USA) and regression using an adequate calibration curve. The permeability (P) of glucose was calculated using the following Equation (2):

(2) $$-\ln\left(1-\frac{2C_{t}}{C_{0}}\right)=\frac{2A}{V}\times P\times t$$

where C_t_ is the concentration in the receptor compartment at time t, C_0_ is the initial concentration in the donor compartment, V is the solution volume in the two compartments, and A is the effective area of permeation.

The diffusion coefficient (D) of glucose across the membrane was calculated according to Fick’s law of diffusion, as follows (Equation (3)):

(3) $$D=\frac{V_{1}V_{2}}{V_{1}+V_{2}}\times \frac{h}{A}\times \frac{1}{t}ln\left(\frac{C_{f}-C_{i}}{C_{f}-C_{t}}\right)$$

where D is the diffusion coefficient (cm^2^ s^−1^); C_i_ and C_f_ are the initial and final concentrations, respectively, and C_t_ is the concentration of solute in the receptor side at time t (mol L^−1^); V_1_ and V_2_ correspond to the volume of the liquid in the donor compartment and in the receptor compartment (cm^3^), respectively; h is the thickness of the membrane (cm); and A is the effective diffusion area of the membrane (cm^2^).

The partition coefficient (K_d_) is defined as a measure of the solubility of glucose in the hydrogel. The partition coefficient for the system was calculated as follows (Equation (4)):

(4) $$K_{d}=\frac{P\times h}{D}$$

where P is the permeability, h is the thickness of the membrane, and D is the diffusion coefficient.

### 3.5. Biological Assessment of Developed Fucoidan-Based Hydrogel

#### 3.5.1. Cell Culture

A human pancreatic cell line (1.1B4HP) was cultured in RPMI 1640 medium (Invitrogen, MA, USA) supplemented with 10% fetal bovine serum (FBS) (Invitrogen, USA) and 1% antibiotic/antimycotic (A/B) (Invitrogen, USA) at a temperature of 37 °C and 5% CO_2_ until achieving 90% confluence. The medium was refreshed every 2–3 days.

#### 3.5.2. Cell Encapsulation

Before the encapsulation procedure, the 1.1B4HP cells (passage 36) were washed with sterile DPBS followed by centrifugation at 300× *g* for 5 min to form a pellet, and the supernatant was discarded. The suspension of 1.1B4HP cells at a concentration of 1.5 × 10^6^ cells mL^−1^ was added to Aga and AgaFu solutions and gently mixed at 37 °C. The obtained suspension (cells:Aga/AgaFu solution) was then cast into appropriate molds, and the hydrogels were progressively formed until room temperature was reached. Subsequently, the hydrogels were removed from molds and replenished with DPBS to enable complete gel formation. After repeated washings, the cell-laden hydrogels were cultured in RPMI 1640 medium for further in vitro studies.

#### 3.5.3. Metabolic Activity, Proliferation, and Morphology

After each of the predetermined periods (1, 3, and 7 days), the samples were washed with DPBS, and the MTS (3-(4,5-dimethylthiazol-2-yl)-5-(3-carboxymethoxyphenyl)-2-(4-sulfophenyl)-2*H*-tetrazolium) assay, DNA quantification, live/dead assay, and assessment of cell morphology, adhesion, and distribution were performed, as described below.

Metabolic activity assay: The metabolic activity of 1.1B4HP cells encapsulated in hydrogels was determined using the MTS assay. Samples were incubated with an MTS solution prepared using a 1:5 ratio of MTS reagent and DMEM without phenol red and 1% ATB solution for 3 h at 37 °C. The optical density (O.D.) was read at 490 nm on a multiwell microplate reader (Bio-Tek Instruments, San Jose, CA, USA).

dsDNA quantification: dsDNA quantification was performed after each experimental time point using the PicoGreen Quantification Kit (Invitrogen Corporation, MA, USA), according to the manufacturer’s instructions, in a lysed cell suspension obtained after osmotic and thermal shocks. Fluorescence was read in a microplate reader (Bio-Tek Instruments, US) at an excitation of 485/20 nm and emission of 528/20 nm.

Live/dead assay: The cellular viability was assessed using the live/dead assay (calcein AM/propidium iodide (PI) staining). Briefly, the cell-laden Aga and AgaFu hydrogels were incubated for 10 min with 2 μL calcein AM (1 mg mL^−1^, Molecular Probes, Invitrogen, USA) and 1 μL PI (1 mg mL^−1^, Molecular Probes, Invitrogen, USA) in 1 mL of DPBS protected from light. The hydrogels were washed with DPBS to remove residual staining and visualized by CLSM (TCS SP8, Leica, Wetzlar, Germany). Image J was used to count the number of live and dead cells, and Equation (5) was used to calculate the percentage of viable cells:

(5) $$Viability=\frac{\%Livecells}{\%Livecells+\%Deadcells}\times 100$$

#### 3.5.4. Cell Morphology

Adhesion, morphology, and distribution of 1.1B4HP cells encapsulated into AgaFu hydrogels were analyzed by CLSM. The cells in Aga and AgaFu hydrogels were fixed with 10% formalin for 30 min and then blocked with 3% bovine serum albumin (BSA) for 30 min. The structures were permeabilized with 0.1% Triton X-100 for 5 min and incubated with phalloidin-TRITC for 20 min at room temperature, followed by washing with DPBS and staining with 5 µg mL^−1^ DAPI (2-(4-amidinophenyl)-1*H*-indole-6-carboxamidine) for 30 min. Fluorescence images from the stained constructs were obtained by CLSM (TCS SP8, Leica Wetzlar, Germany).

### 3.6. Statistical Analysis

Statistical analysis of the data was performed using the Shapiro–Wilk normality test, one-way ANOVA, and non-parametric Kruskal–Wallis and Dunn´s multiple comparison tests, using Graph Pad Prism 7.0. Results are presented as mean ± standard deviation (SD), and the significance level between groups was set for * *p* < 0.1, ** *p* < 0.01, and *** *p* < 0.001.

## 4. Conclusions

In this work, AgaFu hydrogels were obtained through the combination of agarose and fucoidan, two well-known natural-origin polymers, using the thermo-gelation technique at 37 °C. Combining agarose and fucoidan at different concentrations using temperature variations, we have obtained hydrogels with tunable mechanical properties, stability, and diffusion coefficients. Moreover, we were also able to modulate cell viability and promote the organization of HP cells into pseudo-islets. Comparing all the conditions, we concluded that AgaFu 3b hydrogel formulation presented a suitable mesh size of the gel that contributed to a good diffusion of molecules, suggesting higher nutrient permeability. Furthermore, these AgaFu 3 hydrogels revealed sustained viability of encapsulated 1.1B4HP pancreatic cells during the studied culture time (up to 7 days), and these cells seemed to self-organize towards the formation of pseudo-islets.

Overall, we have provided a simple methodology to foster the development of new fucoidan-based structures, and we envisage its biomedical application for cell culture matrices in diabetes treatment strategies.

## Figures and Tables

**Table 1:** Summary of formulations used in the preparation of Aga and AgaFu hydrogels.

Formulation	Polymer Solution Concentration (%)		Hydrogel
	Agarose (Aga)	Fucoidan (Fu)	Aga:Fu Ratio
Aga 3	3	-	-
Aga 5	5	-	-
Fu 3	-	3	-
Fu 5	-	5	-
AgaFu 3a	1.2	3	4:10
AgaFu 3b	1.5		5:10
AgaFu 3c	2.1		7:10
AgaFu 5a	2	5	4:10
AgaFu 5b	2.5		5:10
AgaFu 5c	3.5		7:10

**Table 2:** Relative atomic composition (O, C, and S) of the samples obtained by XPS data quantification and O and S ratios as a function of carbon (data presented as mean ± standard deviation).

Composition %	O	C	S	O/C	S/C
Fu	35.29 ± 0.20	61.52 ± 0.21	3.19 ± 0.05	0.57	0.052
Aga 3	42.95 ± 0.57	56.88 ± 0.06	0.16 ± 0.05	0.75	0.002
Aga 5	47.14 ± 1.04	52.49 ± 1.05	0.38 ± 0.09	0.90	0.007
AgaFu 3a	40.66 ± 0.49	55.06 ± 0.54	4.28 ± 0.12	0.74	0.081
AgaFu 3b	42.77 ± 0.49	53.43 ± 0.52	3.79 ± 0.08	0.80	0.070
AgaFu 3c	45.70 ± 0.59	51.39 ± 0.62	2.91 ± 0.09	0.89	0.057
AgaFu 5a	46.06 ± 0.84	52.26 ± 0.86	1.68 ± 0.13	0.88	0.032
AgaFu 5b	31.93 ± 0.65	66.96 ± 0.67	1.09 ± 0.08	0.48	0.016
AgaFu 5c	54.22 ± 0.84	45.13 ± 0.85	0.65 ± 0.10	1.20	0.014

**Table 3:** Permeability (**P**), diffusion (**D**), and partition (**K_d_**) coefficients of glucose on Aga 3, Aga 5, AgaFu 3, and AgaFu 5 hydrogels with different agarose:fucoidan weight ratios (a (4/10), b (5/10), and c (7/10)).

Samples	P (10^−6^ cm s^−1^)	D (10^−5^ cm^2^ s^−1^)	K_d_
Aga 3	8.03	8.35	0.02
AgaFu 3a	24.36	1.24	0.45
AgaFu 3b	9.91	4.05	0.05
AgaFu 3c	9.94	2.07	0.11
Aga 5	2.38	0.35	0.38
AgaFu 5a	9.90	0.68	0.32
AgaFu 5b	3.99	1.14	0.10
AgaFu 5c	10.61	0.82	0.28

## Data Availability

The data that support the findings of this study are provided in the text or available from the corresponding author upon reasonable request.
